# Limits to Dihydrogen Incorporation into Electron Sinks Alternative to Methanogenesis in Ruminal Fermentation

**DOI:** 10.3389/fmicb.2015.01272

**Published:** 2015-11-18

**Authors:** Emilio M. Ungerfeld

**Affiliations:** INIA Carillanca, Instituto de Investigaciones AgropecuariasTemuco, Chile

**Keywords:** methanogenesis inhibition, rumen, dihydrogen, meta-analysis, thermodynamics, kinetics

## Abstract

Research is being conducted with the objective of decreasing methane (CH_4_) production in the rumen, as methane emissions from ruminants are environmentally damaging and a loss of digestible energy to ruminants. Inhibiting ruminal methanogenesis generally results in accumulation of dihydrogen (H_2_), which is energetically inefficient and can inhibit fermentation. It would be nutritionally beneficial to incorporate accumulated H_2_ into propionate or butyrate production, or reductive acetogenesis. The objective of this analysis was to examine three possible physicochemical limitations to the incorporation of accumulated H_2_ into propionate and butyrate production, and reductive acetogenesis, in methanogenesis-inhibited ruminal batch and continuous cultures: (i) Thermodynamics; (ii) Enzyme kinetics; (iii) Substrate kinetics. Batch (*N* = 109) and continuous (*N* = 43) culture databases of experiments with at least 50% inhibition in CH_4_ production were used in this meta-analysis. Incorporation of accumulated H_2_ into propionate production and reductive acetogenesis seemed to be thermodynamically feasible but quite close to equilibrium, whereas this was less clear for butyrate. With regard to enzyme kinetics, it was speculated that hydrogenases of ruminal microorganisms may have evolved toward high-affinity and low maximal velocity to compete for traces of H_2_, rather than for high pressure accumulated H_2_. Responses so far obtained to the addition of propionate production intermediates do not allow distinguishing between thermodynamic and substrate kinetics control.

## Introduction

Methanogenesis is the main electron sink in ruminal fermentation. Metabolic hydrogen ([H]) released in fermentation is transferred to methanogens mainly as H_2_ and incorporated into CH_4_. Methanogenesis allows carbohydrates to be fermented to a more oxidized product, acetate, instead of ethanol or lactate, which results in greater microbial ATP generation (Wolin et al., 1997). However, the release of CH_4_ to the atmosphere, mainly through eructation and respiration, represents a loss of energy for ruminants, and is also a cause of climate change, as CH_4_ is a potent greenhouse gas (Eckard et al., 2010; Martin et al., 2010).

Because CH_4_ emissions from ruminants are energetically inefficient and environmentally damaging, research has been conducted on the inhibition of ruminal methanogenesis. There are published reports both of *in vitro* (e.g., Bauchop, 1967; Anderson et al., 2003) and *in vivo* (e.g., McCrabb et al., 1997; Kung et al., 2003; Mitsumori et al., 2012) experiments in which CH_4_ production was dramatically decreased using different chemical inhibitors. Metabolic hydrogen management is central to ruminal energetic efficiency. It is desirable to re-direct reducing equivalents that are not incorporated into CH_4_ toward sinks that are nutritionally useful to the host animal, like volatile fatty acids (VFA). Inhibiting methanogenesis diminishes the production of acetate, which is associated with the release of [H], and favors incorporation of [H] into propionate, resulting in a typical acetate to propionate shift (Janssen, 2010). However, in a meta-analysis of methanogenesis inhibition in *in vitro* experiments, incorporation of [H] into propionate production was small for batch culture compared to what the decrease in CH_4_ production would stoichiometrically allow, and on average non-existent in continuous culture (Ungerfeld, 2015).

Butyrate production from hexoses results in net release of [H], but, relative to acetate, butyrate production releases less [H] per mol of hexose fermented. Inhibiting methanogenesis could thus be theoretically expected to cause an acetate to butyrate shift. However, there was no overall increase in the incorporation of [H] into butyrate production in batch or continuous cultures associated to the decrease in CH_4_ production (Ungerfeld, 2015).

Reductive acetogens inhabit the rumen (Leedle and Greening, 1988; Henderson et al., 2010), but reductive acetogenesis is not a [H] sink in the typical ruminal fermentation because methanogenesis lowers H_2_ pressure below the thermodynamic threshold for acetogens to grow (Kohn and Boston, 2000). If methanogenesis is inhibited, addition of reductive acetogens can decrease H_2_ accumulation (Le Van et al., 1998; Lopez et al., 1999).

**Figure 1:**
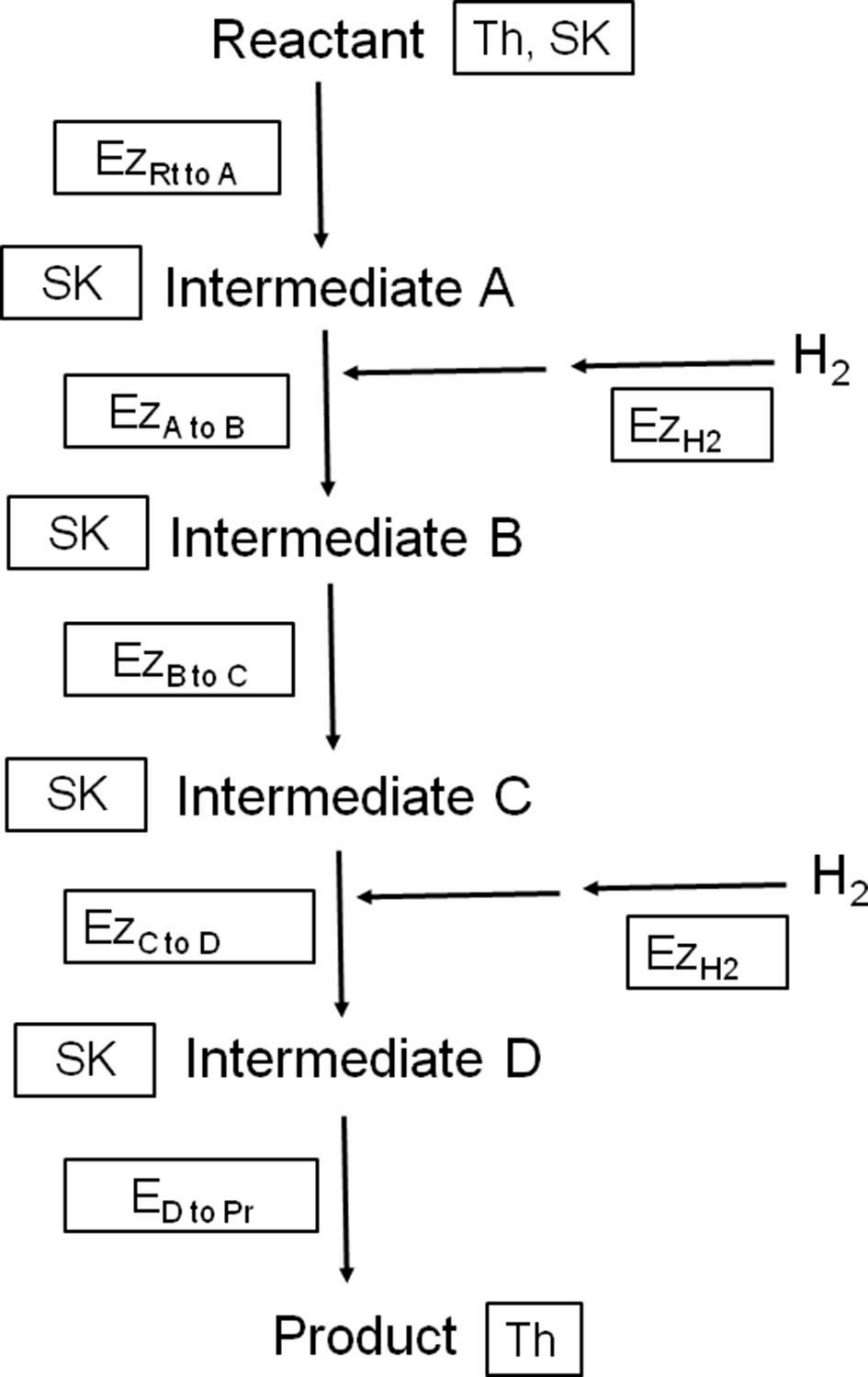
**Illustration depicting three different means of physicochemical control of the rate of a fictitious H_2_-incorporating biochemical pathway starting with a reactant (Rt) that is converted into four intermediates A, B, C and D, and finishing with a product (Pr)**. Th, thermodynamic control, which depends on the activities of the reactant and product; Ez, enzyme kinetics control, which depends on the activity of the most limiting enzyme. Depicted in the diagram are two hydrogenases (Ez_*H*2_) and five enzymes catalyzing conversions between carbon compounds (Ez_*xtoy*_); SK, substrate kinetics control, which depends on the concentration of the reactant or the most limiting intermediate and is not affected by product accumulation.

Propionate and butyrate, and acetate formed through reductive acetogenesis, are nutritionally useful [H] sinks to ruminants as energy and carbon sources. Propionate is also the main source of glucose for ruminants. A question of importance is therefore, what are the physicochemical limitations to the incorporation of accumulated H_2_ into propionate and butyrate formation, and reductive acetogenesis, in the methanogenesis-inhibited ruminal fermentation. The rate and extent of any biological process can be controlled by enzyme or substrate kinetics, or thermodynamics (Figure 1), and understanding the physicochemical control of H_2_ incorporation can help in the design of efficient strategies of manipulation of [H] flows. The present analysis calculates thermodynamic limits of the incorporation of accumulated H_2_ into propionate and butyrate production and reductive acetogenesis, and discusses possible control mechanisms of these processes in the methanogenesis-inhibited fermentation.

## Methods

### Databases

Databases of *in vitro* mixed ruminal batch and continuous cultures were used to calculate the Gibbs energy change (Δ*G*) of the incorporation of H_2_ into propionate and butyrate production and reductive acetogenesis. The present meta-analysis included part of the batch and continuous culture databases used in Ungerfeld (2015). However, only experiments that reported total gas or CO_2_ production were used, in order to calculate CO_2_ partial pressure, and eventually Δ*G* of the H_2_-incorporating processes in which CO_2_ was involved. If total gas production was reported, but CO_2_ production or molar percentage were not, CO_2_ molar percentage was calculated as the difference between total gas and the sum of CH_4_ and H_2_.

The rest of the criteria used for inclusion of experiments were the same as in Ungerfeld (2015):

- Initial headspace was H_2_-free and formic acid or formate salts were not included as substrate;
- The experiment included a methanogenesis-uninhibited control treatment and results for CH_4_, H_2_, and VFA in the control treatment were reported;
- At least one treatment or level within a treatment resulted in a 50% or greater decrease in CH_4_ production relative to the control. This requirement increases variation in the regressor (methanogenesis inhibition) and decreases the proportion of variation in Δ*G* associated to the experiment effect, resulting in greater power to test the hypotheses of responses of Δ*G* to methanogenesis inhibition;
- Treatments within experiments consisting in combinations of methanogenesis inhibitors and fermentation intermediates or their isomers or analogs (malate, fumarate, crotonate, butynoic acid, or 3-butenoic acid) were discarded, as intermediates whose concentration was not reported could affect the thermodynamics of the process being studied;

Some batch culture studies that did not report net VFA production were not used in Ungerfeld (2015) meta-analysis but were included in the present study, because only metabolites final concentrations, but not their net production, is necessary to estimate Δ*G*.

Incubations including different treatments that were run simultaneously and included the same uninhibited control treatment were considered to be one experiment. The batch culture database comprised a total of 109 treatment means from 17 experiments in nine published studies (Table S1). The continuous cultures database comprised a total of 43 treatment means from eight experiments in six peer-reviewed published studies (Table S2).

### Rationale and calculations

Gibbs energy changes of the incorporation of H_2_ into propionate and butyrate production and reductive acetogenesis, were calculated for batch cultures at the end point of the incubations, and for continuous cultures at steady state. Inhibition of methanogenesis *in vitro* generally decreased or did not affect the estimated amount of fermented hexoses (Ungerfeld, 2015). For the present analysis, Δ*G* of H_2_ incorporation into propionate and butyrate production and reductive acetogenesis was estimated assuming that amount of hexoses fermented did not change i.e., accumulated H_2_ was not to be incorporated into pathways of additional hexoses being fermented (e.g., additional propionate production from hexoses).

Interconversions among VFA have been experimentally demonstrated (Ungerfeld and Kohn, 2006). Therefore, [H] in accumulated H_2_ in the methanogenesis-inhibited fermentation could be incorporated into propionate formation through acetate being converted to propionate:

(1) $${\text{CH}}_{3}{\text{COO}}^{-}+{\text{CO}}_{2}+3{\text{H}}_{2}\to {\text{CH}}_{3}{\text{CH}}_{2}{\text{COO}}^{-}+2{\text{H}}_{2}O$$

An acetate to propionate shift with an implied decrease in H_2_ accumulation could also occur if there was a decrease in hexoses fermented to acetate compensated with a concomitant increase in hexoses fermented to propionate i.e., a replacement of ½ × 4 [2H] in the products of Equation (2) with 2 [2H] in the reactants of Equation (3):

(2) $$\begin{array}{l} ½({\text{C}}_{6}{\text{H}}_{12}{\text{O}}_{6}+2{\text{H}}_{2}\text{O}\to 2{\text{CH}}_{3}{\text{COO}}^{-}+2{\text{H}}^{+}\\ \;+2{\text{CO}}_{2}+4[2\text{H}]) \end{array}$$

(3) $${\text{C}}_{6}{\text{H}}_{12}{\text{O}}_{6}+2[2\text{H}]\to 2{\text{CH}}_{3}{\text{CH}}_{2}{\text{COO}}^{-}+2{\text{H}}^{+}+2{\text{H}}_{2}\text{O}$$

The incorporation of two extra pairs of reducing equivalents in half of Equation (2) is stoichiometrically equivalent to incorporation of 2 H_2_ into propionate production (Equation 4):

(4) $$2[\text{2H}]\to {\text{2H}}_{\text{2}}$$

Such a shift of acetate to propionate could occur intra- and/or extracellularly. Some ruminal bacteria can produce propionate from hexoses, whereas others produce succinate or lactate as intermediates, which are then excreted and converted to propionate by other bacteria (Stewart et al., 1997). An intracellular shift from acetate to propionate or its intermediates occurs when a cell produces less acetate and derives part of fermentation-produced [H] from H_2_ and formate to propionate, or succinate or lactate production. For example, the acetate to succinate ratio produced by *Ruminococcus flavefaciens* decreases when grown in pure culture, in comparison to when grown in co-culture with a methanogen (Latham and Wolin, 1977).

Associated to the shift from acetate to propionate occurring when replacing roughages with concentrates (Janssen, 2010), there are changes in the microbial community composition (Petri et al., 2013). The observed shift from acetate to propionate with concentrate supplementation is the result of both changes in the fermentation profile of individual cells and changes in microbial populations, but is currently impossible to determine the contribution of each component.

Production of propionate from hexoses in the typical, uninhibited ruminal fermentation is obviously thermodynamically favorable (Δ*G* modeled by Kohn and Boston, 2000). Inhibiting methanogenesis results in accumulation of H_2_ and reduced cofactors (Hino and Russell, 1985), which would make propionate production from hexoses even more thermodynamically favorable. Therefore, for the present Δ*G* calculation on the incorporation of accumulated H_2_ into propionate production, only incorporation of H_2_ into acetate conversion to propionate as depicted in Equation (1) was considered. In any case, because Δ*G* is a state function, net thermodynamic consequences for a cell or the entire fermentation system of a shift from acetate to propionate through less [H] generated by Equation (2) and more [H] incorporated by Equation (3) as H_2_ (Equation 4) would be equal to Equation 1.

Production of butyrate from hexoses results in a net release of [2H], although [2H] release per mol of hexose is lesser compared to acetate production (Equation 2 vs. Equation 5):

(5) $${\text{C}}_{6}{\text{H}}_{12}{\text{O}}_{6}\to {\text{CH}}_{3}{\text{CH}}_{2}{\text{CH}}_{2}{\text{COO}}^{-}+{\text{H}}^{+}+2{\text{CO}}_{2}+2[2\text{H}]$$

Conversion of acetate to butyrate occurs in the rumen (Ungerfeld and Kohn, 2006) and could theoretically be a mechanism to incorporate [H] in accumulated H_2_ into VFA:

(6) $$2{\text{CH}}_{3}{\text{COO}}^{-}+{\text{H}}^{+}+2{\text{H}}_{2}\to {\text{CH}}_{3}{\text{CH}}_{2}{\text{CH}}_{2}{\text{COO}}^{-}+2{\text{H}}_{2}\text{O}$$

Alternatively, and analogous to propionate production, a decrease in hexoses fermented to acetate compensated by a concomitant increase in hexoses fermented to butyrate would also result in implicit incorporation of accumulated H_2_ into butyrate production, as part of [H] released from acetate production would instead be used to produce butyrate. The net thermodynamic consequences in Δ*G* that result from a combination of Equations (2, 4, 5) are depicted by Equation (6) above.

For the present analysis, Δ*G* of incorporation of accumulated H_2_ into propionate and butyrate were therefore calculated from the incorporation of H_2_ in the conversion of acetate to propionate (Equation 1) or butyrate (Equation 6).

Gibbs energy change for H_2_ incorporation into reductive acetogenesis was calculated as:

(7) $$2{\text{CO}}_{2}+4{\text{H}}_{2}\to {\text{CH}}_{3}{\text{COO}}^{-}+{\text{H}}^{+}+2{\text{H}}_{2}\text{O}$$

Müller, 2003

Gibbs energy changes at standard chemical conditions (Δ*G*°) and 298 K (25°C) for Equations (1, 6, 7) were calculated from Gibbs energy changes of formation of individual compounds (Δ*G*${}_{f}^{{}^{\circ}}$) (Kohn and Boston, 2000; Karadagli and Rittmann, 2007). Gibbs energy changes so calculated were then adjusted to a ruminal temperature of 39°C through the Van't Hoff equation (Kohn and Boston, 2000). Gibbs energy changes at standard chemical conditions and 39°C were subsequently adjusted to actual *in vitro* ruminal fermentation conditions using the activities of soluble metabolites and dissolved gases (Karadagli and Rittmann, 2007). Activities were calculated as the product of concentrations by activity coefficients. Activity coefficients of dissolved CO_2_ and H_2_ were assumed to be equal to unity. Activity coefficients of charged ions in Equations (1, 6, 7) were calculated according to the Davies equation (Hamer, 1968):

$$\text{log}\gamma =z^{\text{2}}\text{A}\sqrt{I}/\left(1+\sqrt{I}\right)+0.2\text{A}I$$

where γ is the activity coefficient (molar scale), *z* is the charge of the ion, *I* is the molar ionic strength of the solution, and A is a molar parameter calculated as:

$$\text{A}=1.825\times 10^{6}/{\left(T\epsilon \right)}^{3/2}$$

where *T* is the absolute temperature (312 K) and ϵ is the dielectric constant of the solvent [73.82 for water at 311 K according to Hamer (1968)].

Extracellular ionic strength was calculated as the sum of ionic strength provided by the medium buffer and macrominerals, and VFA. For batch cultures, ionic strength was calculated from the initial ion concentration provided by the buffer and macrominerals and 0.15 M for the inoculum (Kohn and Boston, 2000). It is acknowledged that a net uptake of ions would take place as cultures grew and that release of CO_2_ initially present in the buffer as HCO${}_{3}^{-}$ would consume equimolar amounts of H^+^ released from VFA formed in fermentation. In turn, CO_2_ released from fermentation would result in H^+^ release from carbonic acid. For continuous cultures, ionic strength was calculated as the sum of ionic strength provided by buffer and macrominerals and VFA at steady state.

Total gas pressure was calculated from the ratio of total gas production to headspace volume in batch cultures, and assumed to be equal to 10^5^ kPa in continuous cultures. Partial pressure for each gas was calculated from the Ideal Gas Law, and dissolved gases concentrations were calculated from Henry's Law (Kohn and Boston, 2000; Janssen, 2010), assuming equilibrium between the liquid and gas phases. Water concentration was assumed to be 50 M (Kohn and Boston, 2000).

In some control treatments, H_2_ was undetectable and reported as equal to zero. In those cases, Δ*G* was calculated assuming a H_2_ concentration equal to the median of controls reporting non-zero H_2_ values for batch (2.35 × 10^−6^ M) or continuous (2.02 × 10^−6^ M) cultures.

Gibbs energy change for reduction of oxidized ferredoxin (Fd_ox_) and NAD^+^ by H_2_ (Buckel and Thauer, 2013) was also estimated for methanogenesis-inhibited and non-inhibited ruminal batch and continuous cultures:

(8) $$\begin{array}{l} {\text{Fd}}_{\text{ox}}+{\text{NAD}}^{+}+2{\text{H}}_{2}\to {\text{Fd}}_{\text{red}}^{2-}+\text{NADH}+3{\text{H}}^{+};\\ \;\Delta G^{{}^{\circ}}=-21\text{ kJ} \end{array}$$

Buckel and Thauer, 2013

For the estimation of Δ*G* for Equation (8) in the normal, methanogenesis non-inhibited fermentation, the NAD^+^/NADH pair was considered to be 95% in the oxidized form (Bennett et al., 2009), and ferredoxin was considered to be 90% in the reduced form (Buckel and Thauer, 2013). For the estimation of Δ*G* in the methanogenesis-inhibited fermentation, the ratios of the reduced to oxidized species of both cofactor pairs were multiplied by a factor equal to 1.79 to account for greater concentration of reduced cofactors under methanogenesis inhibition (Hino and Russell, 1985). This factor is equal to the quotient between 0.70/0.39, which are the NADH/NAD^+^ ratios reported by Hino and Russell (1985) for methanogenesis-inhibited and control ruminal incubations, respectively.

For the estimation of Δ*G* for Equation (8), H^+^ concentration was assumed to be equal to 10^−7^ M for an intracellular pH of 7. Concentration of H_2_ in methanogenesis non-inhibited fermentation was assumed to be equal to the median of controls treatments reporting non-zero H_2_ values for batch (2.35 × 10^−6^ M) and continuous (2.02 × 10^−6^ M) cultures. Concentration of H_2_ under H_2_ accumulation was estimated by regressing the response of dissolved H_2_ to methanogenesis inhibition in batch and continuous cultures and calculating the predicted H_2_ concentration for 100% methanogenesis inhibition.

### Regressions

The responses of calculated Δ*G* for the conversions of acetate to propionate (Equation 1) and butyrate (Equation 6), and for reductive acetogenesis (Equation 7), against the inhibition of methanogenesis expressed as a percentage of CH_4_ production in control treatments, were regressed separately for batch and continuous cultures as:

$$\Delta G_{ij}=\text{intercept}+exp_{i}+{\text{B}}_{1}x_{ij}+{\text{B}}_{2}x_{ij}^{2}+{\text{b}}_{i}x_{ij}+residual_{ij}$$

where *exp*_*i*_ is the fixed effect of the experiment i, B_1_ and B_2_ are fixed linear and quadratic regression coefficients, respectively, of regressor *x* %CH_4_ production inhibition, b_*i*_ is the fixed effect of experiment i on the linear coefficient of *x* (i.e., the interaction between methanogenesis inhibition and the experiment effect), and *residual*_*ij*_ is the residual of the ith experiment at the jth level, with *residual* assumed to be independent and normally distributed. Fixed, rather than random, effect of the experiment was used because the objective of the analysis was to understand how methanogenesis inhibition affects Δ*G* of H_2_ incorporation into propionate, butyrate and reductive acetogenesis within the existing analyzable universe of information, rather than building models to predict Δ*G* for new *in vitro* experiments. Significance was declared at *p* < 0.05 and tendencies at 0.05 ≤ p ≤ 0.10. Non-significant interactions and quadratic effects (*p* > 0.10) were removed, although if removing a non-significant term increased corrected Akaike Information Criterion and/or Bayesian Information Criterion, this term was left in the model.

In meta-analysis, weighting treatment means by the reciprocal of their standard errors (1/SEM) scaled to one is recommended to obtain maximum likelihood estimates while maintaining the original scale of the data (Sauvant et al., 2008). However, SEM of Δ*G* are not calculable from reported data, as they are a logarithmic function of metabolites concentrations, with individual VFA concentrations in turn calculated as the product of total VFA and corresponding molar percentages, each variable with their reported SEM. Instead, reciprocal of SEM of total VFA concentration was chosen as the weighting variable.

Plots of residuals against predicted values were examined. Outliers and influential observations were identified as in Ungerfeld (2015) through examining studentized residuals, leverage (hat) values, and Cook's distances. Potential influential outliers were identified as treatment means with (1) a studentized residual > |t_*n*−*k*_| (=2 SEM), where k was the number of parameters and n the number of treatment means used to fit the regression; (2) a leverage value larger than 2k/n (Belsey et al., 1980); (3) a Cook's distance greater than the 50th percentile of an F_*k, n*−*k*_ distribution.

JMP® 11.0.0 was used for all statistical analyses.

## Results

### Acetate conversion to propionate

**Figure 2:**
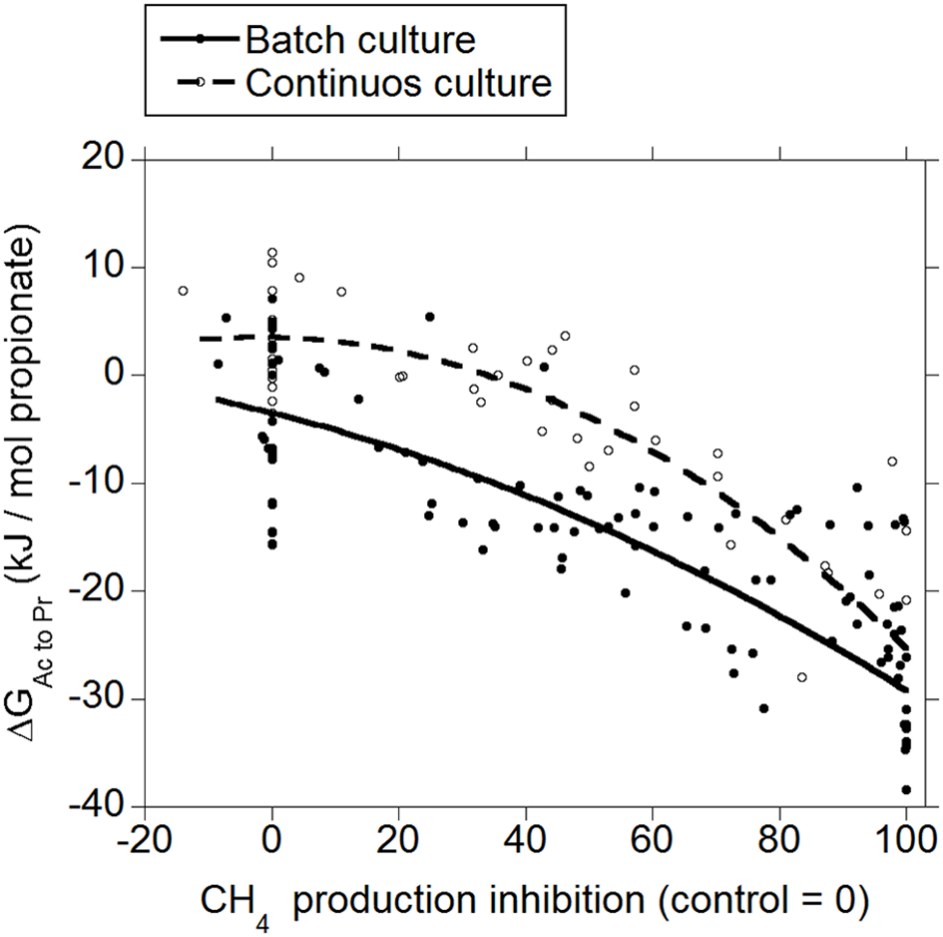
**Response of Gibbs energy of acetate conversion to propionate (Δ*G*_*Ac to Pr*_) to the inhibition of CH_4_ production expressed as a percentage of CH_4_ production decrease relative to control treatments**. Treatment means are adjusted by the effect of their experiment. Batch culture: *y* = −1.03 (±2.12; *p* = 0.63) + *exp* (*p* < 0.001) − 0.25 (±0.029; *p* < 0.001) *x* − 0.0011 (±0.00062; *p* = 0.075) (*x* − 46.6)^2^ + *exp* × *x* (*p* < 0.001); *R*^2^ = 0.93 (*p* < 0.001); Continuous culture: *y* = 9.68 (±2.14; *p* < 0.001) + *exp* (*p* < 0.001) − 0.27 (±0.032; *p* < 0.001) *x* − 0.0028 (±0.0010; *p* = 0.011) (*x* − 46.7)^2^ + *exp* × *x* (*p* = 0.032); *R*^2^ = 0.85 (*p* < 0.001).

Both in batch and in continuous culture, inhibiting CH_4_ production decreased Δ*G* for the incorporation of H_2_ into the conversion of acetate to propionate (*p* < 0.001; Figure 2), with an interaction with the experiment effect (*p* < 0.05). There was a tendency to a quadratic response in batch culture (*p* = 0.075), and a significant quadratic response in continuous culture (*p* = 0.011).

### Acetate conversion to butyrate

**Figure 3:**
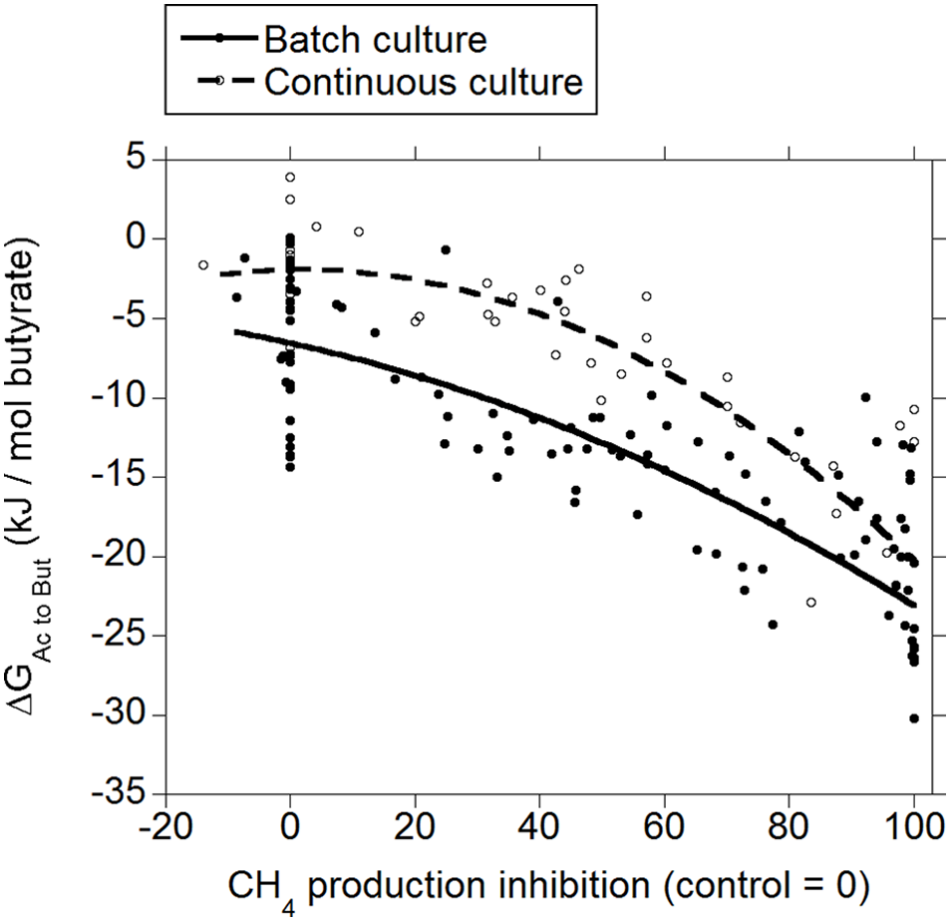
**Response of Gibbs energy of acetate conversion to butyrate (Δ*G*_*Ac to But*_) to the inhibition of CH_4_ production expressed as a percentage of CH_4_ production decrease relative to control treatments**. Treatment means are adjusted by the effect of their experiment. Batch culture: *y* = −4.80 (±1.50; *p* = 0.002) + *exp* (*p* < 0.001) − 0.16 (±0.020; *p* < 0.001) *x* − 0.00079 (±0.00044; *p* = 0.074) (*x* − 46.6)^2^ + *exp* × *x* (*p* < 0.001); *R*^2^ = 0.92 (*p* < 0.001); Continuous culture: *y* = 2.21 (±1.50; *p* = 0.15) − 0.17 (±0.022; *p* < 0.001) *x* − 0.0019 (±0.00072; *p* = 0.016) (*x* − 46.7)^2^ + *exp* × *x* (*p* = 0.030); *R*^2^ = 0.87 (*p* < 0.001).

Inhibiting CH_4_ production decreased Δ*G* for the incorporation of H_2_ into the conversion of acetate to butyrate both in batch and in continuous culture (*p* < 0.001; Figure 3), with an interaction with the experiment effect (*p* < 0.05). There was a tendency to a quadratic response in batch culture (*p* = 0.074), and a significant quadratic response in continuous culture (*p* = 0.016).

### Reductive acetogenesis

**Figure 4:**
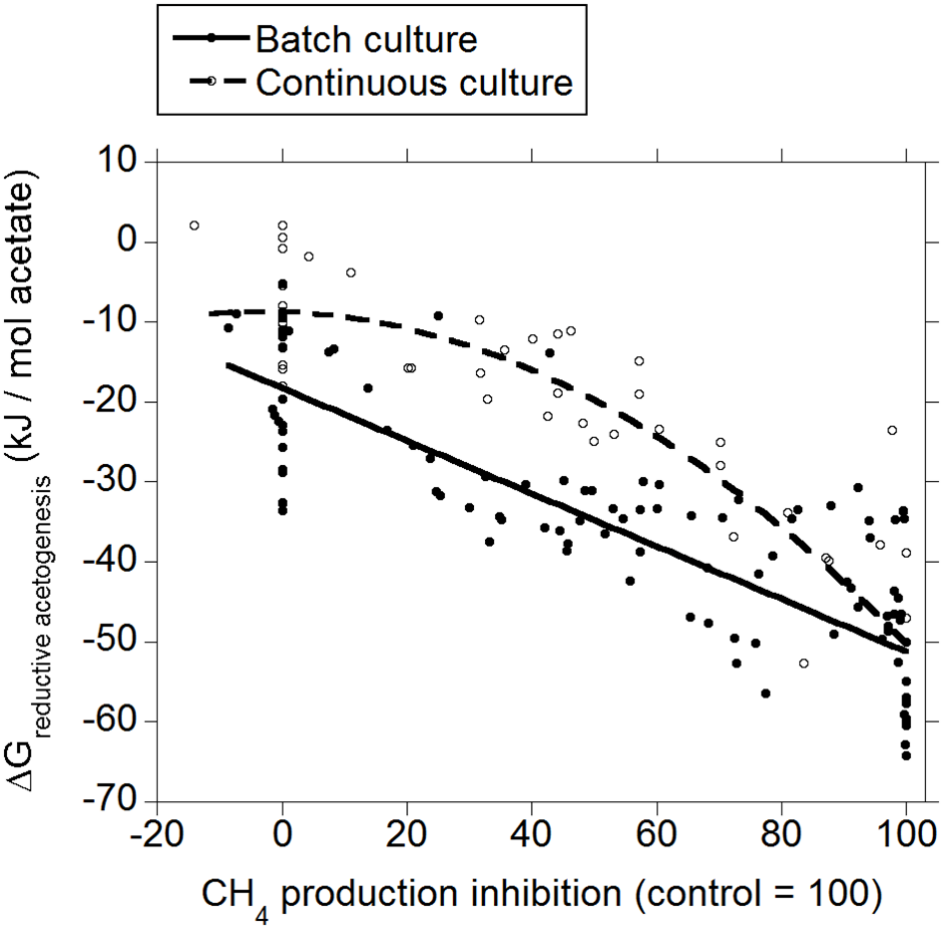
**Response of Gibbs energy of reductive acetogenesis (Δ*G*_*reductive ace to genesis*_) to the inhibition of CH_4_ production expressed as a percentage of CH_4_ production decrease relative to control treatments**. Treatment means are adjusted by the effect of their experiment. Batch culture: *y* = −18.2 (±2.43; *p* < 0.001) + *exp* (*p* < 0.001) − 0.33 (±0.038; *p* < 0.001) *x* + *exp* × *x* (*p* < 0.001); *R*^2^ = 0.93 (*p* < 0.001); Continuous culture: *y* = −0.16 (±2.97; *p* = 0.96) − 0.39 (±0.044; *p* < 0.001) *x* − 0.0039 (±0.0014; *p* = 0.011) (*x* − 46.7)^2^ + *exp* × *x* (*p* = 0.045); *R*^2^ = 0.85 (*p* < 0.001).

Both in batch and continuous culture, there was a decrease in Δ*G* of reductive acetogenesis as methanogenesis was inhibited (*p* < 0.001; Figure 4), with an interaction with the experiment effect (*p* < 0.05). In continuous culture, the response was quadratic (*p* = 0.011).

## Discussion

In the normal, methanogenesis-uninhibited ruminal fermentation, H_2_ production from reduced cofactors is thermodynamically feasible because H_2_ concentration is kept very low through rapid removal mainly by methanogenesis (Janssen, 2010). In the methanogenesis-inhibited ruminal fermentation, H_2_ removal is smaller but greater ratio of reduced to oxidized cofactors (Hino and Russell, 1985) thermodynamically allows H_2_ accumulation. The present analysis addresses the question of what are the physicochemical limitations to the incorporation of [H] in accumulated H_2_ into propionate or butyrate production, or reductive acetogenesis, when methanogenesis is inhibited. Three physicochemical potential limitations to H_2_ incorporation into propionate and butyrate production and reductive acetogenesis will be discussed:

- A thermodynamic limitation would occur if propionate or butyrate production, or reductive acetogenesis, reached thermodynamic equilibrium at the concentrations of metabolites observed in the *in vitro* cultures and according Equations (1, 6, 7), respectively;
- An enzyme kinetics limitation could be one such as insufficient numbers of microorganisms possessing the genes encoding for the necessary enzymes, expression of those genes, enzyme activity regulation, or enzymes *k*_*m*_ and *v*_*max*_;
- A substrate kinetics limitation would be an insufficient rate of provision of electron-accepting carbon compounds to incorporate accumulated H_2_ into the desired pathways.

### Thermodynamics

The typical range in the profile of VFA in ruminal fermentation has been proposed to result from thermodynamic equilibrium (Ungerfeld and Kohn, 2006). Calculated Δ*G* for acetate conversion to propionate or butyrate in the ruminal fermentation with functional methanogenesis (zero methanogenesis inhibition) were not different from or very close to zero (Figures 2, 3; Table 1), which agrees with the proposition of thermodynamic control of the VFA profile. Reductive acetogenesis has been estimated to be slightly thermodynamically favorable but close to equilibrium in the typical ruminal fermentation (Kohn and Boston, 2000), in accord to current Δ*G* estimation in batch culture at zero methanogenesis inhibition; in continuous culture at zero methanogenesis inhibition, reductive acetogenesis Δ*G* was estimated to be not different from zero (Figure 4; Table 1).

**Table 1:** **Predicted Δ*G* for acetate conversion to propionate and butyrate and for reductive acetogenesis for batch and continues cultures at 0 and 100% methanogenesis inhibition**.

**Process**	**System**	**Methanogenesis inhibition (%)**	***ΔG* (kJ / mol VFA)**	**95% confidence interval**
Acetate to propionate	Batch	0	−3.42	−10.3 to 3.42
		100	−29.2	−42.7 to −15.7
	Continuous	0	3.57	−5.15 to 12.3
		100	−25.3	−41.8 to −8.79
Acetate to butyrate	Batch	0	−6.50	−12.8 to −0.16
		100	−23.1	−34.5 to −11.7
	Continuous	0	−1.93	−7.99 to 4.27
		100	−20.2	−31.8 to −8.59
Reductive acetogenesis	Batch	0	−18.2	−23.0 to −13.4
		100	−51.2	−63.5 to −38.9
	Continuous	0	−8.67	−20.8 to 3.48
		100	−50.2	−73.1 to −27.3

As CH_4_ production was inhibited and H_2_ accumulated, H_2_ incorporation into reductive acetogenesis, propionate production, and butyrate production, in that order, became thermodynamically more favorable (Figures 2–4; Table 1). The decreases in Δ*G* of each process relative to each other reflect their relative stoichiometries of H_2_ incorporation. Likewise, the interactions between methanogenesis inhibition and the experiment effect reflect the variation among experiments in the response in H_2_ accumulation to methanogenesis inhibition, as H_2_ accumulation is the main driving force for Δ*G* decrease due both to the stoichiometry of H_2_ incorporation compared to carbon-containing compounds, and to the relative magnitude of the changes in H_2_ concentration in comparison to the rest of the metabolites. Variation among methanogenesis-inhibition experiments with regard to H_2_ accumulation has been previously discussed (Ungerfeld, 2015), thus the interactions between methanogenesis inhibition and the experiment effect will not be addressed herein.

Minimal predicted Δ*G* at 100% methanogenesis inhibition (i.e., with maximal H_2_ accumulation) was slightly negative for incorporation of H_2_ into propionate and butyrate production and reductive acetogenesis both in batch and continuous cultures (Table 1). It has been postulated that bacterial metabolism can occur at Δ*G* very close to thermodynamic equilibrium (Jackson and McInerney, 2002); however, Δ*G* calculations herein do not consider the utilization of Δ*G* in ATP generation. Given a Δ*G* of ATP hydrolysis of −40 to −50 kJ/ mol, and that thermodynamic efficiency of ATP generation from ADP and phosphate in the energy-conserving process can approach 100% (Thauer et al., 1977; Voet and Voet, 1995), it appears from Table 1 that if 1 mol of ATP per mol of acetate was generated by reductive acetogenesis, the process would be close to equilibrium, and that incorporation of H_2_ into both propionate and butyrate production from acetate could not generate a Δ*G* negative enough to generate 1 mol of ATP per mol of propionate or butyrate, respectively. However, ATP is generated through transmembrane ion gradient-driven phosphorylation both in reductive acetogenesis and in fumarate reduction to succinate in propionate's randomizing pathway (Russell and Wallace, 1997; Müller, 2003), and less than one ATP may be generated per pair of reducing equivalents incorporated (Reddy and Peck, 1978; Kröger and Winkler, 1981). Furthermore, a minimum ATP generation for maintenance and growth may not be required: reductive acetogens (Joblin, 1999), and propionate producers could obtain most of their energy from sugar fermentation, and perhaps supplement it with a small amount of ATP obtained from ion gradient-driven phosphorylation from reductive acetogenesis and acetate conversion to propionate, in each case. In principle therefore, incorporation of accumulated H_2_ into propionate formation or reductive acetogenesis, although close to equilibrium, may perhaps have been thermodynamically feasible in the experiments analyzed. Estimated thermodynamic feasibility of reductive acetogenesis under H_2_ accumulation *in vitro* agrees with a decrease in H_2_ verified when reductive acetogens have been added to methanogenesis-inhibited ruminal incubations (Nollet et al., 1997; Le Van et al., 1998; Lopez et al., 1999).

Predicted Δ*G* of H_2_ incorporation into butyrate production at 100% methanogenesis inhibition was also slightly negative (Table 1). However, butyrate production involves the generation of 1 mol of ATP per mol of butyrate through substrate level-phosphorylation. This requirement seems to make the incorporation of additional H_2_ into butyrate production with acetate as the carbon donor thermodynamically unfeasible. However, ATP generation associated to butyrate production through ion gradient-driven phosphorylation recently proposed for the genus *Butyrivibrio*, may allow these bacteria greater ATP generation than what has been previously thought for this pathway (Hackmann and Firkins, 2015), and perhaps enable incorporation of H_2_ in the conversion of acetate to butyrate.

One should be cautious about the thermodynamic calculations herein presented. Limitations to estimations of Δ*G* have been discussed by Maskow and von Stockar (2005). Metabolite concentrations, and most significantly H_2_, are not uniform in microbial ecosystems and gradients exist (Boone et al., 1989). In particular, lack of equilibrium between gaseous and dissolved H_2_ can result in large differences between Δ*G* estimated assuming equilibrium and actual Δ*G* calculated from the aqueous phase concentration of gases (Hackmann, 2013). Working with ruminal batch cultures, Wang et al. (2014) reported that H_2_ was supersaturated in the liquid phase with respect to the headspace, and there was no relationship between concentration of dissolved H_2_ and gaseous H_2_. Thus, actual Δ*G* are likely lower than Δ*G* estimated assuming equilibrium between gaseous and dissolved H_2_, making the processes discussed likely more feasible than has been herein estimated.

Rumen contents can be described as a more heterogeneous environment compared to *in vitro* cultures, due both to greater solid particle size and solids to fluid ratio. Greater heterogeneity likely creates more microenvironments with H_2_ gradients *in vivo* than *in vitro*, implying that H_2_-incorporating pathways could be more thermodynamically favorable *in vivo* close to H_2_ production microenvironments compared to the present estimations from *in vitro* results. On the other hand, gases accumulate to supra-atmospheric pressures in batch cultures compared to continuous cultures and the rumen. This could give batch cultures a thermodynamic edge compared to continuous cultures and *in vivo* fermentation with respect to H_2_ incorporation; perhaps this causes Δ*G* of H_2_ incorporation into propionate and butyrate production and reductive acetogenesis to be slightly more favorable in batch than in continuous culture (Figures 2–4), due to numerically greater response in H_2_ accumulation to methanogenesis inhibition (Ungerfeld, 2015).

### Enzyme kinetics

#### Propionate production

**Figure 5:**
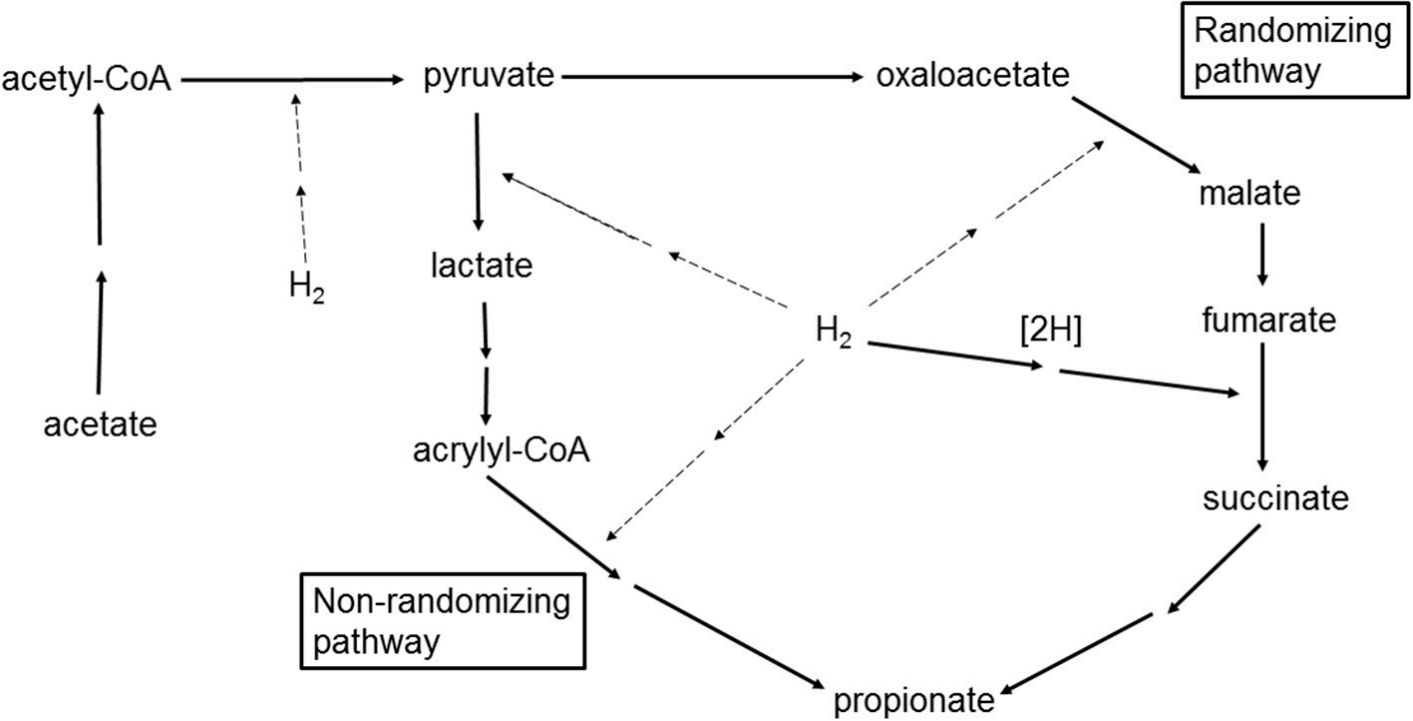
**Incorporation of H_2_ in the conversion of acetate to propionate through the randomizing and non-randomizing pathways**. Two sequential arrows represent multiple reactions condensed for simplicity. Carbon dioxide, water, and HS-CoA are omitted for simplicity. Solid arrows represent known or demonstrated reactions. Dashed arrows represent putative pathways of H_2_ incorporation.

Enzyme-limited capacity of the mixed ruminal microbiota to produce propionate can be caused by insufficient activities of enzymes catalyzing conversions between carbon compounds or by insufficient activity of hydrogenases (Figure 5). With regard to enzymes catalyzing conversions between carbon compounds, Denman et al. (2015) recently found an increase in the genes encoding for most of the enzymes of propionate's randomizing pathway when methanogenesis was inhibited in goats.

With respect to hydrogenases activity, H_2_ incorporation in the reduction of fumarate to succinate has been demonstrated in ruminal bacteria (Henderson, 1980; Asanuma et al., 1999), but H_2_ incorporation into the other reduction steps in Figure 5 has not been shown for ruminal fermentation. The question that will be discussed next is if the mixed ruminal microbiota could potentially incorporate H_2_ at a fast enough rate to avoid H_2_ accumulation when CH_4_ production is inhibited, if H_2_ incorporation into additional propionate production from acetate is indeed thermodynamically feasible.

Recent research efforts have focused on understanding *in vivo* changes in the ruminal microbial community composition when methanogenesis is inhibited. Mitsumori et al. (2012) reported an increase in *Prevotella* sp. and *Clostridium aminophilum*. *Prevotella* sp. produce propionate (Stewart et al., 1997) and are often the most abundant organisms in the rumen (Stevenson and Weimer, 2007; Bekele et al., 2010; Stiverson et al., 2011). Moreover, *P. ruminicola* can incorporate H_2_ into propionate production (Henderson, 1980). The increase in the abundance of *Prevotella* sp. agreed with an increase in propionate concentration (Mitsumori et al., 2012). Shinkai et al. (2012) also found an increase in *P. ruminicola*, along with propionate producer *S. ruminantium* when methanogenesis was inhibited, and variable changes in other propionate and succinate producers. However, along the increases in propionate concentration and abundance of propionate producers (Mitsumori et al., 2012; Shinkai et al., 2012), and genes encoding for enzymes of propionate's randomizing pathway (Denman et al., 2015) observed with *in vivo* methanogenesis inhibition, there still has been persistent long-term H_2_ accumulation (Trei et al., 1971, 1972; Kung et al., 2003; Mitsumori et al., 2012). Therefore, the changes occurring in these microbial populations and presumably the kinetic capacity to produce propionate, do not seem to be sufficient to take up all of the accumulated H_2_.

Broudiscou et al. (2014) inoculated batch cultures with continuous cultures vastly differing in H_2_ content in their headspaces. Adding H_2_ to the batch cultures headspace actually decreased propionate molar proportion when the inoculum came from a low-H_2_ continuous culture, and did not affect it when the inoculum came from high-H_2_ continuous cultures. These results suggests no long-term adaptation of the mixed microbiota growing in those continuous culture fermenters to incorporate elevated H_2_ into propionate production, both because of H_2_ accumulation occurring in the continuous culture fermenters themselves, and because of lack of response in propionate to H_2_ in batch cultures inoculated with high-H_2_ continuous cultures. It should be noted though that lack of adaptation to elevated H_2_ in this experiment may partially reflect loss of microbial diversity in continuous cultures (Johnson et al., 2009).

Changes in the ruminal microbial community composition and metabolism as a response to supplementation with intermediates of propionate production have also been studied. Mao et al. (2008) reported that feeding disodium fumarate to goats induced *Succinivibrio dextrinisolvens*-related organisms, which would likely reduce the added fumarate to succinate. Supplementation with disodium fumarate did not affect the abundance of a *P. ruminicola*-related organism. Zhou et al. (2012) found that feeding disodium fumarate to sheep decreased succinate-producers *Fibrobacter succinogenes* in ruminal solids, and *R. flavefaciens* in ruminal fluid in one trial and caused erratic results in a longer term trial. However, the abundance of organisms such as *Prevotella* spp., *Selenomonas* spp., or *Succinivibrio* spp., was not evaluated in that study. In pure culture, several succinate and propionate-producing bacteria increased fumarate reductase activity when grown in a fumarate-containing medium (Asanuma and Hino, 2000).

Several ruminal bacteria produce formate as a means of disposing [H] (Stewart et al., 1997). In the mixed ruminal fermentation, formate is an intermediate whose concentration is kept low because formate produced is rapidly consumed mainly in CH_4_ formation (Hungate et al., 1970; Asanuma et al., 1998). If methanogenesis is inhibited, formate often accumulates (Asanuma et al., 1998; Ungerfeld, 2015). Therefore, it is important to find ways to incorporate also formate into [H] sinks useful to the host animal. Most bacterial species that were able to use H_2_ as [H] donor in fumarate reduction to succinate could also use formate, although their affinity for formate was generally lower than for H_2_ (Asanuma et al., 1999).

Addition of a direct-fed microbial would enhance the enzyme kinetics capacity of pathways carried out by the added microbes. For example, Henning et al. (2010) successfully decreased lactate concentration in the rumen of acidotic lambs and steers by dosing a strain of lactate utilizer *Megasphaera elsdenii*, demonstrating that utilization of accumulated lactate was being limited by enzyme kinetics in the control treatments. Mamuad et al. (2014) decreased CH_4_ production in mixed ruminal batch cultures by adding the fumarate-reducing bacterium *Mitsuokella jalaludinii*. Competition for [H] with methanogenesis was therefore in their experiment controlled by enzyme kinetics, but because propionate production was not affected by addition of *M. jalaludinii* and succinate increased only temporarily, it is difficult to conclude which was the competing [H] sink or sinks. On the other hand, addition of propionibacteria *in vivo* was unsuccessful to decrease CH_4_ production or increase propionate molar proportion (Aikman et al., 2011; Vyas et al., 2014a,b, 2015). Vyas et al. (2014a) quantified the added propionibacteria strains throughout the day and concluded that added propionibacteria did not persist long enough in the rumen to cause effects on CH_4_ and propionate. It is also possible that enhancement of enzyme kinetics without accumulated H_2_ did not result in extra propionate if additional propionate production was not enzyme-, but substrate- or thermodynamically limited. Evaluation of the effect of the addition of a propionate or succinate producer in methanogenesis-inhibited *in vitro* systems or *in vivo* would allow understanding if and under which conditions incorporation of accumulated H_2_ into propionate production can be limited by enzyme kinetics.

#### Butyrate production

**Figure 6:**
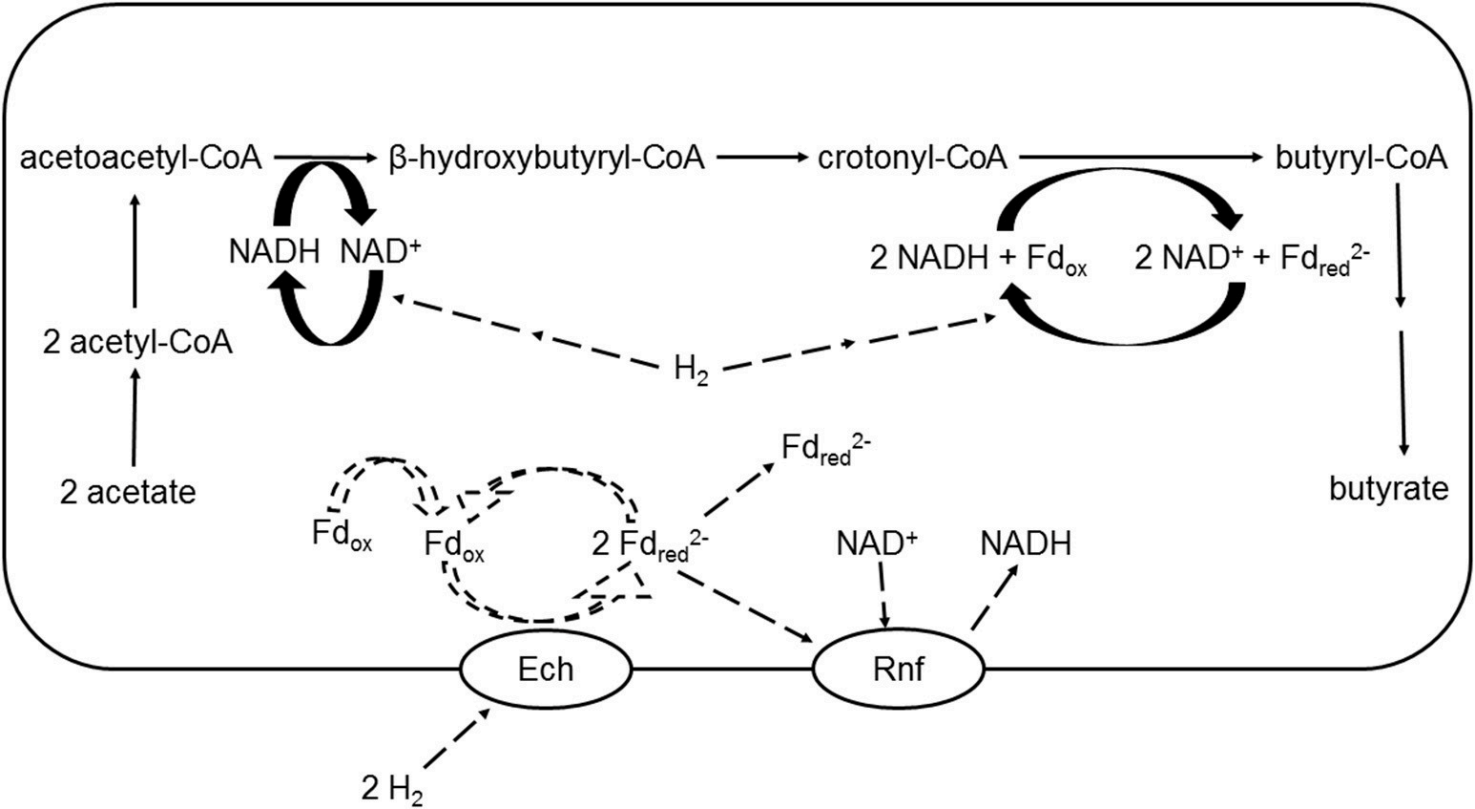
**Upper part: putative incorporation of H_2_ in the conversion of acetate to butyrate in a ruminal *Butyrivibrio* spp. cell; lower part: putative mechanism of electron transfer from H_2_ to NAD^+^ as described by Buckel and Thauer (2013) for non-ruminal organisms: Fd_ox_ + NAD^+^ + 2 H_2_ → Fd${}_{\text{red}}^{2-}+$ NADH + 3 H^+^**. Ech, *Escherichia coli* hydrogenase-3-type hydrogenase transmembrane ion pump; Rnf, *Rhodobacter* nitrogen fixation transmembrane ion pump; Fd_ox_, oxidized ferredoxin; Fd_red_, reduced ferredoxin; Solid arrows represent known or demonstrated pathways. Dashed arrows represent putative H_2_ incorporation and electron transfer reactions. For simplicity, reactions are not balanced for H^+^, and extruded cations, water and HS-CoA are omitted. Adapted from Hackmann and Firkins (2015).

Overall, there was no re-direction of [H] toward butyrate production when methanogenesis was inhibited in batch or continuous culture (Ungerfeld, 2015). The same as propionate, H_2_ incorporation into butyrate production could potentially be limited by insufficient activities of enzymes catalyzing the conversion between carbon compounds or by insufficient activities of hydrogenases to incorporate elevated H_2_ (Figure 6). A first question is then if the mixed ruminal microbiota can incorporate H_2_ into butyrate production. Non-ruminal *Clostridium kluyveri* was shown to use H_2_ as [H] donor for the reduction of acetoacetate to ß-hydroxybutyrate in butyrate production (Cohen and Cohen-Bazire, 1950). In ruminal *B. fibrisolvens* D1 (Miller and Jenesel, 1979) and *Megasphaera* (formerly *Peptostreptococcus*) *elsdenii* (Baldwin and Milligan, 1964), the [H] donors for butyrate production were NADH and NADPH. Also in *M. elsdenii*, Whitfield and Mayhew (1974) showed that the reduction of crotonyl-CoA to butyryl-CoA was coupled to the oxidation of D-lactate to pyruvate. In the above described findings in ruminal bacteria, the reductions of acetoacetate to ß-hydroxybutyrate and of crotonyl-CoA to butyryl-CoA would therefore be coupled to intracellular electron transfer reactions rather than to incorporation of H_2_. Baldwin and Milligan (1964), however, showed that [H] could be transferred from H_2_ to NAD^+^ using ferredoxin in *M. elsdenii*.

In a recent comprehensive analysis of the energy metabolism of the genus *Butyrivibrio*, Hackmann and Firkins (2015) reported that 47 out of 62 *Butyrivibrio* spp. genomes possessed genes encoding for all six subunits of *Escherichia coli* 3-type hydrogenase (Ech), a transmembrane ion pump that transfers electrons in reduced ferredoxin to protons to form H_2_. A question of interest would be if under elevated H_2_, Ech could catalyze the reverse reaction and reduce oxidized ferredoxin using H_2_ (Figure 6). Interestingly, all 47 *Butyrivibrio* spp. genomes that possessed genes encoding for Ech also had genes encoding for ion pump *Rhodobacter* nitrogen fixation Rnf, which catalyzes the reduction of ferredoxin by NADH (Hackmann and Firkins, 2015). Perhaps, if H_2_ could donate electrons to reduce ferredoxin when H_2_ accumulation made this reaction thermodynamically favorable, reduced ferredoxin could then reduce NAD^+^ in a reaction catalyzed by Rnf. NADH thus generated would be available as a direct [H] donor for butyrate production from acetate, with the net result of [H] indirectly donated by H_2_ for butyrate production (Equation 10, Figure 6):

(8) $$\begin{array}{l} {\text{Fd}}_{\text{ox}}+{\text{NAD}}^{+}+2{\text{H}}_{2}\to {\text{Fd}}_{\text{red}}^{2-}+\text{NADH}+3{\text{H}}^{+};\\ \;\Delta G^{{}^{\circ}}=-21\text{ kJ} \end{array}$$

Buckel and Thauer, 2013

(9) $$\begin{array}{l} \text{crotonyl}-\text{CoA}+{\text{Fd}}_{\text{ox}}+2\text{NADH}\to \text{butyryl}-\text{CoA}\\ \;+2{\text{NAD}}^{+}+{\text{Fd}}_{\text{red}}^{2-} \end{array}$$

Buckel and Thauer, 2013

(10) $$\begin{array}{l} \text{crotonyl}-\text{CoA}+2{\text{Fd}}_{\text{ox}}+2{\text{H}}_{2}+\text{NADH}\to \text{butyryl}-\text{CoA}\\ \;+\;2{\text{Fd}}_{\text{red}}^{2-}+3{\text{H}}^{+}+{\text{NAD}}^{+} \end{array}$$

Equation 10 = Equation 8 + Equation 9

It can be estimated that the reduction of ferredoxin and NAD^+^ with H_2_ as [H] donor (Equation 8; Buckel and Thauer, 2013) may be thermodynamically favorable even in the non-methanogenesis inhibited ruminal fermentation without H_2_ accumulation (Table 2).

**Table 2:** **Estimated Gibbs energy changes for the reduction of oxidized ferredoxin and NAD^+^ with dihydrogen (Equation 8)**.

***In vitro* system**	**Methanogenesis**	**Predicted dissolved H_2_ (M)**	***ΔG* (kJ)**
Batch	Non-inhibited	2.35 × 10^−6^	−60,151
Batch	100% inhibited	1.11 × 10^−4^	−77,112
Continuous	Non-inhibited	2.02 × 10^−6^	−59,366
Continuous	100% inhibited	2.54 × 10^−5^	−69,468

It remains to be studied how gene expression and activity of enzymes involved in the conversion of acetate to butyrate is affected by H_2_, but results so far on the response to acetate addition indicate that the operon encoding for enzymes involved in butyrate production in *B. fibrisolvens* (not including butyryl-CoA/acetyl-CoA transferase and crotonase) is constitutively transcribed (Asanuma et al., 2005). If the expression of the operon encoding for enzymes involved in butyrate production does not respond to H_2_ (or to the ratio of reduced to oxidized cofactors), this would be an indication that an important group of ruminal butyrate producers did not evolve to utilize elevated H_2_ (or perhaps even to incorporate H_2_).

#### Reductive acetogenesis

Reductive acetogenesis is functional and even predominates over methanogenesis in the gastrointestinal tract of some humans and termites, rodents, pigs, the rumen of newborn lambs (Joblin, 1999) and kangaroos (Godwin et al., 2014). Reductive acetogens inhabit the rumen (Leedle and Greening, 1988; Le Van et al., 1998) and, potentially, could utilize H_2_ to reduce CO_2_ to acetate as a source of energy and carbon if the process was thermodynamically feasible. However, the fact that the enzyme kinetics capacity to conduct reductive acetogenesis is present in the rumen does not mean that it is non-limiting in the short- or long-term to incorporate atypically high H_2_ concentrations. An important question would be if the abundance of native ruminal reductive acetogen populations can be increased by favorable thermodynamic conditions in the long term. In such a case, a long term decrease in H_2_ accumulation would be expected. The few *in vivo* results reporting the long-term evolution of accumulated H_2_ in methanogenesis-inhibited rumens over time are contradictory, with some long-term decrease in H_2_ accumulation (Clapperton, 1974; Hristov et al., 2015) or steady state gaseous H_2_ concentration (Kung et al., 2003). Knight et al. (2011) reported a decrease in reductive acetogen numbers in the rumen when CH_4_ production was inhibited with chloroform, which was interpreted as a possible consequence of chloroform toxicity. Mitsumori et al. (2014) reported that, although the total number of reductive acetogen sequences was unaffected by methanogenesis inhibition, the phylogeny of reductive acetogens changed. Recently, Denman et al. (2015) found that methanogenesis inhibition in goats resulted in a decrease in the reads of reductive acetogenic enzymes CO dehydrogenase/acetyl-CoA synthase complex and methyltetrahydrofolate:corrinoid/iron-sulfur protein. Their results do not support previous speculation on reductive acetogenesis partially accounting for the observed decrease in [H] recovery in *in vitro* methanogenesis-inhibited ruminal fermentation (Ungerfeld, 2015).

An alternative to stimulation of native populations of reductive acetogens would be the daily or frequent dosing of a reductive acetogen to rumens also receiving a potent inhibitor of methanogenesis. If the reductive acetogenic microbial additive adapted to the rumen environment and used accumulated H_2_ to reduce carbon dioxide to acetate, a decrease in H_2_ accumulation would be expected, albeit to a H_2_ pressure higher than in the ruminal fermentation with active methanogenesis, due to the higher H_2_-threshold of reductive acetogenesis compared with methanogenesis (Thauer et al., 1977; Cord-Ruwisch et al., 1988). That said, reductive acetogens are not obligate hydrogenotrophs and can ferment sugars (Joblin, 1999), therefore, the actual decrease in H_2_ would depend both on the extent of mixotrophy of the dosed reductive acetogen and the availability of alternative substrates. This approach would require screening of reductive acetogens with the highest *v*_max_ and lowest *k*_*m*_ for H_2_ and suitable to be used as a microbial additive mixed with the feed. The ideal reductive acetogen additive would be an obligate hydrogenotroph, if that could exist naturally, or developed as a genetically-engineered organism with deleted carbohydrate metabolism genes.

A situation somewhat analogous to methanogenesis inhibition occurs in experiments in which gnotobiotic animals are inoculated with methanogens-free microbial communities. Gnotobiotic lambs inoculated with reductive acetogens had more reductive acetogens than control lambs. Also, batch incubations under H_2_ and CO_2_ inoculated with methanogen-free ruminal fluid from gnotobiotic lambs produced more acetate than control incubations inoculated with ruminal fluid from conventional lambs (Fonty et al., 2007). This result could indicate that the reductive acetogenic capacity in gnotobiotic lambs had developed enough to contribute to H_2_ incorporation. However, because *in vivo* H_2_ accumulation was not reported in that study, the extent to what reductive acetogenesis could decrease H_2_ accumulation in these methanogens-free rumens is unknown.

### Substrate kinetics

Tatsuoka et al. (2008) and Ebrahimi et al. (2011) could decrease H_2_ accumulation in methanogenesis-inhibited ruminal batch cultures by adding fumarate or malate. Whilst the addition of a substrate or a fermentation intermediate might remove or decrease the substrate kinetics limitation of a process, it could at the same time make it thermodynamically feasible. This approach, therefore, does not allow distinguishing between substrate kinetics and thermodynamic control.

End product accumulation does not affect substrate kinetics but decreases thermodynamic feasibility. Therefore, adding a fermentation end product could be a more suitable approach to distinguish between substrate kinetics and thermodynamic controls. One could envision an experiment in which the rate of a process responded positively to the addition of an intermediate. If this response was negated by the addition of an equimolar amount of the corresponding end product, we could conclude that the process was thermodynamically limited before any addition; if the response remained unchanged when adding the fermentation end product along with the intermediate, we would conclude that the process had been under substrate kinetics control before any addition.

In principle, being acetate and CO_2_ plentiful in ruminal fermentation, it seems unlikely that the availability of these substrates could limit the incorporation of accumulated H_2_ into propionate or butyrate production from acetate, or reductive acetogenesis.

## Final remarks

The present analysis suggests that the incorporation of accumulated H_2_ in the methanogenesis-inhibited ruminal fermentation into acetate conversion to propionate and reductive acetogenesis, and perhaps into acetate conversion to butyrate, may have not be thermodynamically limited in most of the *in vitro* experiments analyzed. Limitations of the Δ*G* calculations presented include the low number of treatment means used in the continuous culture analyses, and the fact that more than half of the treatment means in the batch culture analyses belonged to one study. Also, H_2_ equilibrium between aqueous phase and headspace was assumed, and extracellular, rather than intracellular, concentrations of metabolites and ionic strength were used in the calculations. Incomplete knowledge on actual ATP generation in the processes considered also adds uncertainty to this conclusion.

Even if the incorporation of accumulated H_2_ into propionate and butyrate production from acetate, and into reductive acetogenesis, was thermodynamically feasible, the capacity to incorporate elevated H_2_ resulting from methanogenesis inhibition may be enzyme-limited because of insufficient *v*_max_ of non-methanogenic H_2_-incorporating enzymes to take up H_2_ at the rates it is produced, even at the lower H_2_ production rate of the methanogenesis-inhibited fermentation. One must also bear in mind that in processes close to equilibrium, the reverse reactions may proceed fast enough so as to decrease the net rate of the forward reaction substantially (Ungerfeld and Kohn, 2006).

The present analysis was conducted based on *in vitro* results, and one aspect of enzyme kinetics in which *in vitro* cultures differ from the rumen is microbial diversity. In *in vitro* cultures, possible lack of enough capacity of non-methanogenic hydrogenotrophs to take advantage of elevated H_2_ may be magnified because of lack of adaptation of key organisms incorporating H_2_ into alternative pathways, as loss of microbial diversity has been reported for both batch (Weimer et al., 2011) and continuous (Johnson et al., 2009) cultures. That said, and as discussed, inhibition of methanogenesis *in vivo* has also consistently resulted in long-term H_2_ accumulation (Trei et al., 1971; Kung et al., 2003; Mitsumori et al., 2012; Hristov et al., 2015).

It may be that ruminal microbiota can shift and adapt to dietary changes, and even unphysiological changes such as ruminal contents exchange between animals, to maintain a similar fermentation pattern (Weimer et al., 2010), but perhaps not to incorporate all [H] spared from methanogenesis into VFA production. If some of the H_2_-incorporating processes analyzed have not reached thermodynamic equilibrium, it could be that ruminal hydrogenases that incorporate H_2_ into processes such as propionate (and perhaps butyrate) production, and reductive acetogenesis, have evolved toward high affinity to compete for H_2_ traces, but at the same time toward low *v*_max_, in the typically low-H_2_ ruminal environment. If the ruminal microbiota has not evolved to respond to methanogenesis inhibition by incorporating H_2_ at high rates, there might be a niche for high-rate H_2_–incorporating microbial additives to engineer fermentation toward desired products when CH_4_ production is inhibited. If the limitation is thermodynamic, there would be no responses to exogenously added microorganisms.

A metabolomics approach could give insights on the physicochemical control of the dynamics of H_2_ incorporation in the rumen, given that *in vivo* studies on methanogenesis inhibition have often measured VFA concentrations, but not actual flows of VFA production. Transcriptomic and proteomic analyses of functional genes involved in fermentation pathways can be valuable approaches to gain insights on enzyme kinetics beyond what has been found so far using genomics, but the ultimate answers about enzyme kinetics control would be provided by enzyme activity assays. *In vitro* and *in vivo* experimental work on understanding the physicochemical control of H_2_ incorporation in ruminal fermentation is recommended.

### Conflict of interest statement

The author declares that the research was conducted in the absence of any commercial or financial relationships that could be construed as a potential conflict of interest.
