# Supplementary material for: Limits to Dihydrogen Incorporation into Electron Sinks Alternative to Methanogenesis in Ruminal Fermentation
Source: Front Microbiol. 2015 Nov 18;6:1272. doi: 10.3389/fmicb.2015.01272 (PMC4649033; doi:10.3389/fmicb.2015.01272)
Supplement: Supplementary file 1 [file Table1.DOCX]

**Table S1**. Studies used in the ruminal batch cultures meta-analysis on the effects of methanogenesis inhibition on metabolic hydrogen sinks.

| Study | Number of experiments | Number of treatment means | Substrate and amount (mg) | Volume (ml) | Inoculum species | Incubation (h) | Inhibitor of CH_4_ production (%maximum decrease in CH_4_ production) |
| --- | --- | --- | --- | --- | --- | --- | --- |
| Ungerfeld *et al.* (2003) | 1 | 4 | roughage, 300 | 50 | bovine | 24 | propynoic acid (76) |
| Lila *et al.* (2004) | 1 | 5 | mixed, 200 | 40 | bovine | 6 | diallyl maleate (76) |
| Mohammed *et al.* (2004a) | 1 | 4 | high-concentrate, 200 | 40 | bovine | 6 | horseradish oil (88) |
| Mohammed *et al.* (2004b) | 1 | 4 | high-concentrate, 200 | 40 | bovine | 6 | iodopropane (97) |
| Ungerfeld *et al.* (2005) | 1 | 7 | roughage, 250 | 50 | bovine | 24 | algal fatty acid (97) |
| Ungerfeld *et al.* (2006) | 4 | 12 | roughage, 400 or 500 | 50 | bovine | 24 | propynoic acid (69), ethyl-2-butynoate (100) |
| Anderson *et al.* (2010) | 1 | 7 | roughage, 200 | 10.1 | bovine | 24 | dimethyl-2-nitroglutarate (97),  2-nitromethylpropionate (98), nitroethane (99) |
| Lin *et al.* (2011) | 1 | 2^a^ | cellulose/starch, 200 | 50 | bovine | 24 | sodium nitrate (79) |
| O'Brien *et al.* (2013) | 6 | 64 | roughage or mixed | 50 | bovine | 24 | lauric (99), linoleic (83) and linolenic acid (100), 2-bromoethanesulphonic acid (57), bromochloromethane (100), pyromellitic diimide (100) |

^a^Treaments with centrifuged ruminal fluid as inoculum excluded.
