# Supplementary material for: Limits to Dihydrogen Incorporation into Electron Sinks Alternative to Methanogenesis in Ruminal Fermentation
Source: Front Microbiol. 2015 Nov 18;6:1272. doi: 10.3389/fmicb.2015.01272 (PMC4649033; doi:10.3389/fmicb.2015.01272)
Supplement: Supplementary file 2 [file Table2.DOCX]

**Table S2**. Studies used in the ruminal continuous cultures meta-analysis on the effects of methanogenesis inhibition on metabolic hydrogen sinks.^a^

| Study | Number of experiments | Number of treatment means | Substrate and amount (g DM/d) | Dilution rate (h^-1^) | Inhibitor of CH_4_ production  (%maximum decrease in CH_4_ production) |
| --- | --- | --- | --- | --- | --- |
| Slyter (1979) | 1 | 6 | roughage, 12.1 | 0.0625 | monensin (33), dichloroacetamide (100), dichloroacetamide + monensin (100), low pH (96), low pH + monensin (98) |
| Stanier and Davies (1981) | 3 | 9 | mixed, 16.7 | 0.0188 to 0.0417 | 2-trichloromethyl-4-dichloromethylene benzo[1,3] dioxin-6-carboxylic acid (87), monensin (53) |
| Dong *et al.* (1997) | 1 | 8 | roughage or high concentrate, 10 | 0.025 | canola oil (44), cod liver oil (57), coconut oil (88) |
| Soliva *et al.* (2004) | 1 | 8 | mixed, 14 | 0.0208 | lauric and myristic acids (70) |
| Klevenhusen *et al.* (2009) | 1 | 8 | roughage or mixed, 15 | 0.0165 | monolaurin (53) |
| Watanabe *et al.* (2010) | 1 | 4 | high concentrate, 12 | 0.0208 | cashew nut shell liquid (70) |

^a^Bovine ruminal contents were used as inoculum in all experiments in continuous cultures.
